# Implementing shared decision-making interventions in breast cancer clinical practice: a scoping review

**DOI:** 10.1186/s12911-023-02263-8

**Published:** 2023-08-23

**Authors:** Natalia Oprea, Vittoria Ardito, Oriana Ciani

**Affiliations:** Centre for Research on Health and Social Care Management (CeRGAS), SDA Bocconi School of Management, Milan, 20136 Italy

**Keywords:** Implementation, Patient decision aids, Shared decision-making, Breast cancer, PRISM framework

## Abstract

**Background:**

Shared decision-making (SDM) is a collaborative process whereby patients and clinicians jointly deliberate on the best treatment option that takes into account patients’ preferences and values. In breast cancer care, different treatment options have become available to patients in the last decade. Various interventions, including patient decision aids (PtDAs), have been designed to promote SDM in this disease area. This study aimed at investigating the factors that influence the successful adoption and implementation of SDM interventions in real-world healthcare delivery settings.

**Methods:**

A scoping review of scientific and grey literature was conducted for the period 2006–2021 to analyse the support for SDM interventions and their adoption in breast cancer clinical practice. The interpretation of findings was based on the Practical, Robust Implementation and Sustainability Model (PRISM) for integrating research findings into practice.

**Results:**

Overall, 19 studies were included for data synthesis, with more than 70% published since 2017. The availability of SDM tools does not automatically translate into their actual use in clinical settings. Factors related to users’ co-creation, the clinical team’s attitude and knowledge, organisational support and regulatory provisions facilitate the adoption of SDM interventions. However, overlooking aspects such as the re-organisation of care pathways, patient characteristics, and assigning of resources (human, financial, and facilities) can hinder implementation efforts.

**Conclusions:**

Compared to the mounting evidence on the efficacy of SDM interventions, knowledge to support their sustained implementation in daily care is still limited, albeit results show an increasing interest in strategies that facilitate their uptake in breast cancer care over time. These findings highlight different strategies that can be used to embed SDM interventions in clinical practice. Future work should investigate which approaches are more effective in light of organisational conditions and external factors, including an evaluation of costs and healthcare system settings.

**Supplementary Information:**

The online version contains supplementary material available at 10.1186/s12911-023-02263-8.

## Background

Progress in breast cancer (BC) care has contributed to transforming this condition into a chronic rather than a life-threatening illness [1, 2]. Different treatment options have become available to patients, ranging from endocrine-based to surgical therapies. However, deciding the appropriate treatment is burdensome, no less because in some cases, the evidence on outcomes is uncertain, while in others, the options presented are valued differently by patients. In such a complex medical decision-making context, not only is a participative and deliberative process with the patient preferable, but decision support techniques can be an effective approach to satisfy patients’ decisional needs [3, 4].

The growing attention to higher quality in cancer care has recognised shared decision-making (SDM) as an important attribute of patient-centred care [5]. One way to pursue SDM is to adopt decision-support interventions, including patient decision aids (PtDAs). PtDAs promote patients’ engagement by providing evidence-based information about different options and associated benefits and harms, thus enabling congruence between decisions and personal values. In this review, decision support interventions, SDM tools and PtDAs are used interchangeably to refer to structured practices that enable the process of SDM.

Recent updates in international clinical guidelines for BC care emphasise the need to foster the adoption of SDM approaches in clinical practice [6, 7]. Consequently, a growing number of studies have been published recently on SDM supporting tools across countries and clinical practices. While the lion’s share of research has focused on evaluating the efficacy of PtDAs, little importance has been given to the use of SDM interventions in clinical settings. To date, real-world implementation of SDM and PtDAs is still a challenge [8]. In this context, a scoping review was suitable to synthesise the strategies available for the effective implementation of SDM interventions, with specific attention to the influences of the patient, team, organisational, and system-level factors. The goal of the study was to map and analyse the empirical evidence concerning factors that support the implementation of SDM interventions in the delivery of BC healthcare.

Through the lens of the implementation science frameworks and tools, one can investigate what works, for whom, and how, when introducing innovations related to SDM in clinical practice [9]. To integrate the key features for successful SDM interventions, predictors of uptake and diffusion, and appropriate implementation strategies, we used the PRISM framework, a Practical, Robust Implementation and Sustainability Model for translating research knowledge into practice [10]. PRISM favoured our purpose of adopting an integrated approach to implementation by highlighting determinants at multiple levels and recognising their inter-relationship, rather than merely discussing barriers and enablers for uptake. Identifying barriers and enablers does not elucidate whether they are the actual determinants of implementation and their real importance for adoption (e.g. hypothetical barriers and enablers) [11].

## Methods

A scoping review of the scientific and grey literature was performed according to the updated methodological guidance [12] and PRISMA-ScR guidelines for scoping reviews [13] (see Additional File 1 for PRISMA-ScR checklist). Scoping reviews are a type of knowledge synthesis that follows a systematic approach to map relevant concepts, theories, sources, and knowledge gaps in a certain area by extensively identifying, reviewing, and synthesising the evidence available in the literature [14].

### Search strategy and eligibility criteria

The search was performed in three electronic databases: PubMed, Web of Science, and Scopus, covering a timespan between January 2006 and October 2021. The starting point was set in 2006 in concomitance with the creation of the International Patients Decision Aids Standards (IPDAS) [4], which provide a core set of quality criteria for the development of PtDAs. Other studies were identified through ‘snowballing’ techniques, using the references list of relevant published reviews or manual browsing.

The search strategy was built on two content areas, namely SDM and BC. We decided to focus on SDM tools and strategies for BC patients only – as opposed to an array of cancer types – in light of both the unique characteristics of BC patients (e.g. mostly women, decisions on several different aspects beyond treatment, such as the role of fertility prevention) and the availability of multiple clinically effective treatment pathways in this disease area, which has led to abundant literature in this field. Searches were restricted to title and abstract, without filtering the search based on language or country of publication (See Additional File 2 for complete queries). All retrieved articles were imported into a reference manager application.

Empirical studies (randomised control trials, observational and qualitative) illustrating both the *development* and *implementation* of a given SDM intervention were included. Studies formally declared as implementation or hybrid, namely designs with a dual focus on assessing clinical effectiveness and implementation, were considered in our sample [15]. SDM interventions which facilitate treatment decision for patients with a diagnosis of BC were included. We did not restrict studies based on the users (e.g. patients vs. patients & healthcare professionals—HCPs), or delivery method (e.g. paper-based, digital, coaching).

Conversely, studies that focused solely on measuring the efficacy at the patient level and did not investigate development and/or implementation of PtDA components were excluded. Similarly, studies analysing SDM interventions related to screening or preventive therapies for yet-undiagnosed patients and aftercare were excluded. Finally, already published literature reviews, clinical guidelines, or conference abstracts were also excluded, although their references were assessed for potentially relevant studies.

### Study selection, data extraction and analysis

Two researchers (VA, NO) screened the first half of the retrieved titles and abstracts. The second half of the records were independently screened by two reviewers (VA, NO). Titles deemed eligible for full-text reading were assessed in-depth (VA, NO). Disagreements were solved by dialogue with a third researcher (OC). The entire research team read all the studies included in the analysis.

An ad hoc data extraction template was developed, containing information on study identifier, country, study design, type of SDM intervention, the underpinning implementation framework, treatment choice, study setting, and intervention development if available (e.g. participants and methods of engagement). Data were tabulated and summarised through summary descriptive statistics. The template was then supplemented with four PRISM domains, while the taxonomy of Powell and colleagues [16] was used to identify the implementation strategies applicable to each domain. The evidence was interpreted using a narrative approach according to the four components of the PRISM framework.

## Results

**Fig. 1:**
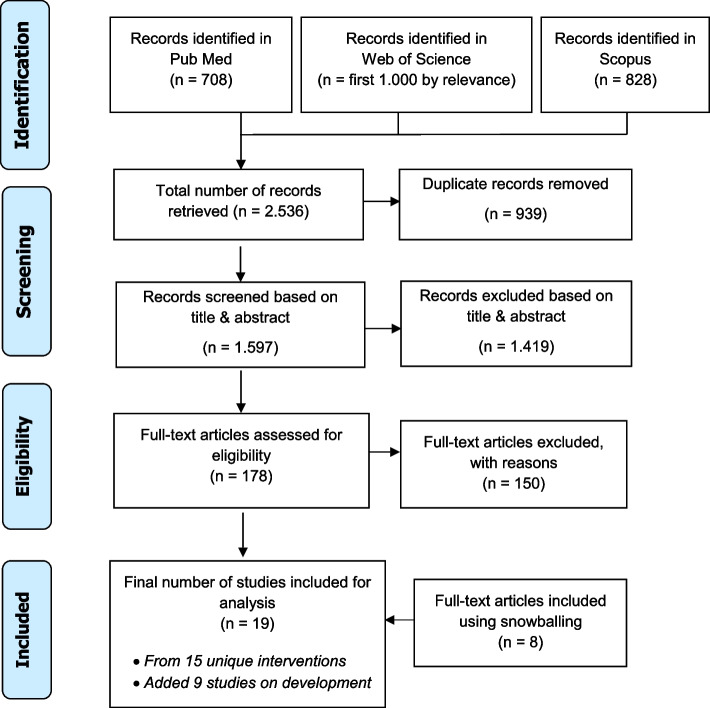
Flow diagram of data searches and source selection

A total of 2.536 papers were identified from the searches. After removing duplicates, the remaining records were screened based on titles and abstracts. Inter-rater agreement (0,86) was measured using the Kappa statistics [17]. A pool of 178 publications was assessed for full-text reading, with eight additional papers included through snowballing search. A total of 19 relevant articles for implementation were finally considered for data extraction and analysis (see Fig. 1 [12]).

### Descriptive overview

The review confirmed the increasing attention to SDM and PtDA adoption over time. In the 2006–2021 timeframe, two-thirds of the studies (74%) were published since 2017. Implementing SDM interventions attracted interest mostly from North America (47%), with eight studies set in the USA and one in Canada, with Europe following suit (42%) with four studies conducted in the Netherlands, two each in the UK and Germany and two in Australia and in partnership with New Zealand (See Table 1).

**Table 1:** Characteristics of all included studies

**Study, year**	**Country**	**Study design**	**Treatment choice**	**SDM intervention**	**Conceptual framework**	**Clinical setting**	**# Participants in implementation study**	**Development of intervention**	
								***Methods used***	***Participants in development***
Berger-Höger et al. 2019	Germany	cRCT	Ductal carcinoma in situ (DCIS)	Booklet; Nurse-led intervention; Structured encounter	Nil reported	16 breast centres	64 patients	Focus groups (FG) and interviews [18]	22 healthy women, 4 breast cancer patients
Burton et al. 2021 [19]	UK	cRCT	Primary endocrine therapy vs surgery, Adjuvant chemotherapy after surgery	Booklet; Web-based PtDA; Risk algorithm	Dose, Reach, Fidelity and Adaptation	8 intervention sites, 8 control sites	82 patients, 10 clinicians	Interviews, FGs [20]	22 women: 14 healthy, 8 patients
van Veenendaal et al. 2021	Netherlands	Pre-post implementation	Various	Implementation programme	Four-level framework for designing implementation strategy	Hospitals: 1 university, 2 teaching, 3 general	22 clinicians, 105 patients	NA	NA
Raphael et al. 2021 [21]	Netherlands	Pre-post implementation	Radiation treatment	Web-based PtDA	Consolidated Framework for Implementation Research (CFIR)	14 radiation oncology centres	181 patients, 78 clinicians	Interviews, think-aloud sessions, live group meetings [22]	Patients, HCPs, patient advocates
Hahlweg et al. 2019 [23]	Germany	Qualitative	Lumpectomy vs. Radiotherapy; Mastectomy	Option grid	Nil reported	1 university hospital	66 patients (observed in 77 encounters)	Cognitive interviews, focus group [23]	9 patients (phase 1), 13 patients, 13 clinicians (phase 2)
Savelberg et al. 2019 [24]	Netherlands	Qualitative	Breast conserving vs Mastectomy	Web-based PtDA	Nil reported	7 regional hospitals	27 HCPs	Interviews, FGs [25]	26 patients, 26 HCPs
Ager et al. 2018 [26]	Australia & New Zealand	Qualitative	Contralateral prophylactic mastectomy	Booklet	Ottawa Decision Support Framework	NA	23 patients, expert panel	Interviews [26]	NA
Schubbe et al. 2021 [27]	USA	Qualitative	Lumpectomy with Radiation vs. Mastectomy	Conversation aids: Option grid, Picture Option Grid	Normalization Process Theory Framework	7 Urban and rural cancer centres	43 patients, 16 surgeons, 14 stakeholders (N = 73)	Community-Based Participatory Research approach [28, 29]	Option Grid: 18 academics and HCPs (initial testing); 53 lay individuals (further testing). Picture Option Grid: 5 community stakeholders (phase 1), 268 patients (phase 2), 15 patients and HCPs (phase 3)
Boateng et al. 2021 [30]	USA	Qualitative	Breast reconstruction	Web-based PtDA	CFIR	2 academic and 1 community health centre	13 patients, 13 clinicians, 9 informatics	NA [31]	40 participants
Belkora et al. 2009 [32]	USA	Qualitative	Radiation vs chemo or hormonal therapy	Video; Booklet	Nil reported	1 academic medical centre	1 patient (case study)	NA	NA
Tollow et al. 2021 [33]	UK	Qualitative	Breast reconstruction	Coach-led intervention; Workbook	Nil reported	5 NHS sites	27 patients (16 from ‘usual care’, 11 from intervention group), 13 HCPs	NA	NA
Silvia et al. 2008 [34]	USA	Qualitative	Various	Video	Nil reported	2 academic medical centres, 9 community hospitals and 1 community oncology centre	Nurses, social workers, patient educators, coordinators	NA	NA
Silvia et al. 2006 [35]	USA	Qualitative	Surgery	Video; Booklet	Nil reported	2 community resource centres, 1 community hospital, 6 academic cancer centres	13 providers and staff	NA	NA
Sherman et al. 2017 [36]	Australia	Qualitative	Breast reconstruction	Web-based PtDA	Nil reported	6 metropolitan- based breast clinics, 3 public, 3 private, and 2 regional private breast clinics	36 patients, 6 HCPs	Focus groups [37]	15 patients (8 had undergone and 7 had not undergone breast reconstruction
Belkora et al. 2015 [38]	USA	Observational study	NA	Coach-led intervention; Booklet	RE-AIM Framework (Reach, Effectiveness, Adoption, Implementation and Maintenance of intervention)	breast cancer centre/clinic	3416 patients	NA	NA
Savelberg et al. 2021 [39]	Netherlands	Observational study	Breast conserving vs Mastectomy	Web-based PtDA	Nil reported	4 regional hospitals	84 patients	Interviews, FGs [25]	26 patients, 26 HCPs
Squires et al. 2019 [40]	Canada	Mixed methods	Contralateral prophylactic mastectomy	Web-based PtDA	Ottawa Decision Support Framework	35 teaching, 4 general hospitals	39 HCPs, 12 patients	NA	NA
Feibelmann et al. 2011 [41]	USA	Mixed-methods	Various	Video; Booklet	Rogers’ theory of innovation diffusion	79 community health centres and private practices	59 HC providers	NA	NA
Bruce et al. 2018 [42]	USA	Survey	Surgery early BC	Web-based PtDA	Replicating Effective Programs framework	1 academic, 1 community clinic	208 patients, 6 surgeons	NA	NA

Ten studies (53%) used qualitative methods, four studies (21%) used experimental methods, such as (clustered) RCTs and pre-post implementation, and the remaining five used observational, survey, or mixed methods design. Most interventions concerned decisions relating to surgery (42%), including contralateral prophylactic mastectomy (CPM), lumpectomy and mastectomy, and breast reconstruction. In seven cases, the interventions incorporated a web-based tool (37%), compared to three using a paper-based one, and four using a booklet and a video, and a mix of web-, paper-based, and coaching sessions (See Table 1).

In terms of clinical settings, 15 out of the 19 analysed studies were carried out in multicentred settings. Seven of these multicentred studies involved a mix of academic and general hospitals, and community or specialised centres. Predominantly, general, regional and community hospitals were utilised in six studies; community (resource) and academic cancer centres in five studies, university and teaching hospitals in three studies, private practices/clinics in two studies, and finally, radiation oncology and metropolitan, rural and urban centres in one study each (See Table 1).

**Fig. 2:**
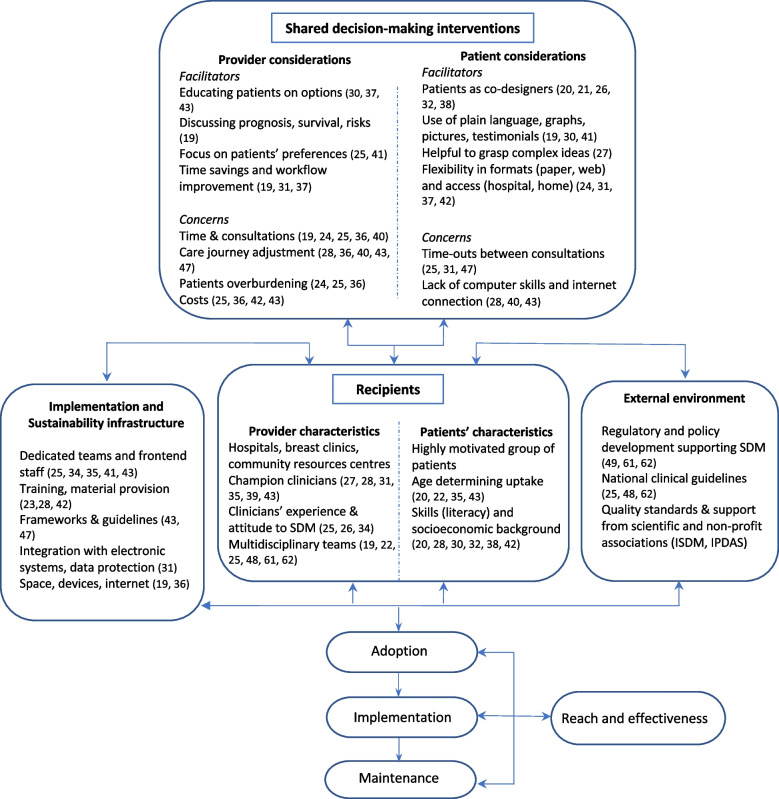
The PRISM Framework categories anticipating implementation factors of SDM  interventions in BC care

In the next paragraphs, we describe the factors that might influence the implementation of interventions at multiple levels using PRISM-relevant domains, summarised in Fig. 2. The four domains of the PRISM implementation model are: patient/provider considerations, recipient characteristics, external environment, and implementation and sustainability infrastructure. In accordance with PRISM, we use the more general category of ‘recipients’ to refer to both organisations (leaders, managers and staff) and patients, whereas ‘user(s)’ refer to a sub-group of recipients, i.e. patients and healthcare providers.

### The intervention: shared decision-making and decision aid use

SDM is defined as a process of collaboration between patients and clinicians in reaching a joint decision about care, involving multiple medically appropriate options [43]. Various decision-support interventions can assist this process (e.g. PtDA, coaches). The PRISM framework considers the perspectives of both patients and providers on such interventions [10]. We examine below these actors’ perspectives on both the development and implementation of PtDA in BC care.

#### Patients’ perspective 

Despite the need to ensure patient-centred care, according to Feldstein and Glasgow [10], patients’ perspective is often diluted by other concerns when developing and delivering healthcare interventions. Our analysis showed that 10 out of 19 analysed studies (see Table 1) gave an account of patients’ involvement in development processes. The majority explicitly consulted patients or cancer survivors in the design and/or pilot testing of the intervention. When patients were not involved, healthy volunteers [20], patient advocates, or other stakeholders were engaged either in the design or testing phases [22, 28, 40].

Early patient engagement is a good predictor of implementation success [44]. Using a systematic and efficient development method can save resources (later in the adoption) and consider hard-to-reach patients (disadvantaged groups, age, etc.). Patients as co-designers of interventions have a twofold role: (i) as experts of their lived experience, conveying needs, concerns and circumstances [31, 37, 45]; and (ii) as evaluators of interventions, by (pilot) testing the comprehension, usability and acceptability of tools [20, 25, 28, 46]. Patients and caregivers participated in the development through interviews and focus groups mainly, as well as surveys [40]. More specifically, early patient participation enabled the discovery of challenges related to communicating prognosis, risks and uncertainty. As a response, the tools were revised using plain and soft language concerning the estimates of personal risks [31], clearer diagrams [20], graphs, pictures or pictographs, followed by text [22, 25], interactive values clarification exercises [26] and patient testimonials [37].

Overall, patients expressed satisfaction regarding the layout, wording and the use of pictorial content or diagrams to visually represent information [19, 29, 40]. These aspects contributed to facilitating patient understanding and maximising the acceptability of the intervention in real-life conditions. Patients felt that these tools enabled them to grasp complex ideas (e.g. CPM not associated with extended survival, in Ager et al. [26]), be more engaged in the decision-making process (for instance when a combination of written and verbal information was used, in Burton et al. [19]) and to focus their mind while handling the information in a moment of high emotional stress [33]. Consultation planning (question listing, audio recording) was found highly valuable by patients for later follow-ups and checking with the treatment plan [32].

Tools accessible from multiple devices and locations (e.g. from home), as often as required were widely preferable for patients [23, 30, 36]. Consequently, distributing web-based versions of PtDAs facilitated implementation [41]. However, while patients were relatively open to various channels of dissemination (e.g. electronic health record, e-mail, mail, patient portal), paper-based material was still highly important [27]. Related to this, different studies found that the lack of computer skills and internet connection might prevent patients from using web-based tools [39, 42]. In summary, a ‘one-size-fits-all’ approach is not feasible; rather, different formats and moments of delivery should be identified, based on patients’ needs and characteristics.

Embedding PtDAs in the clinical pathway can be challenging, as they require finding a balance between their timely use and compliance with treatment guidelines [24], and because patients might need more time before and after clinical encounters or additional consultations to process the diagnosis [30, 47]. Patients expressed a preference for receiving PtDA from their surgeons during the first appointment when the benefits and risks are discussed [27, 39], together with other material following diagnosis [26]. However, the distribution of PtDAs along with other materials (e.g. from charities), should be carefully considered. An evaluation involving older patients emphasised the risk of PtDAs getting lost among other resources, causing information overload in patients [19]. Alternatively, decision-support interventions could be successfully delivered before the oncologist appointment by intern staff [32].

#### Provider’s perspective

Clinicians and other relevant actors (e.g. breast care nurses, specialised researchers, and software engineers) were involved in the PtDA development either individually, through expert panels, or through multi-expert teams. Their engagement was relevant for deciding on the content of the intervention, for instance, by collecting evidence regarding the best treatment for specific conditions [40]. Likewise, HCPs were involved in prototyping and multiple rounds of testing to ensure full agreement on the content [18, 22, 25]. HCPs’ role was crucial in the development phase, not only to inform the PtDA content but most importantly, to generate a sense of ownership and confidence [25, 45], thereby contributing to later buy-in, which ultimately facilitated implementation.

Across the different studies, clinicians had a positive attitude towards SDM interventions, seeing them as useful and helpful in moderating patient-clinician interactions. The key benefits included educating patients about different options available [29, 36, 42], facilitating discussions on prognosis and survival predictions [19], shifting the focus from short- to long-term expectations, for instance, concerning breast reconstruction [33], enabling patients to make more conscious treatment choices [24] on what matters most to them [40]. Features like the centralised, evidence-based content of PtDAs [19, 30] and their interactive nature [36] were also widely appraised by practitioners.

Nevertheless, providers expressed various concerns regarding the translation of these practices into daily care. Clinicians reported difficulties in embedding the use of PtDAs into clinical pathways, including time constraints and length of consultations [19, 24, 35, 39]. Likewise, providers were concerned that PtDAs could generate extra costs without evident returns, and complicate care by confusing patients with too much information [24, 35] or overburdening them [23].

Studies reported that the adjustment of the clinical pathways, for instance by providing time-outs for SDM with patients [47] or making the PtDA fit for surgeons’ consultations, facilitated the successful integration of the interventions [27, 35]. Ideally, the administration of PtDAs was better received after the diagnosis, at the end of consultations [39], before meeting the surgeon or the radiation oncologist [21, 42], and generally before making the final treatment decision [35]. In specific cases such as CPM, surgeons were advised to use a decision-support tool at their initial consultation, informing patients about the lack of oncological benefit of CPM [40]. To be effective, PtDAs were referred to patients either by the surgeons or breast care nurses [36, 41] and by multidisciplinary teams in their report advising the use of PtDAs to clinicians and patients [21].

A concern prevalent among clinicians was the lack of time which might prevent adoption [23, 41]. Nevertheless, various studies found that clinicians, after some experience, were able to normally integrate the tools into their workflow [27], without major changes in the usual care [42], reporting a perceived improvement and time savings due to the use of tools [30]. Other studies found that PtDAs did not seem to alter the length or number of regular consultations [21, 27, 47].

Finally, to overcome the issue of costs and encourage the uptake, interventions or (a set of) copies of PtDAs were made free of charge [24, 35, 41] or web links developed by NGOs, as opposed to commercial organisations, were used for dissemination [42]. Similarly, it was observed that scalability through online distribution favours access and facilitates the use of PtDAs by patients [35, 41]. However, as mentioned earlier, such strategies should be mindful about patients’ skills and technology access.

### Recipients

The extent to which a programme or intervention will be successfully adopted depends on how well it suits its target population. The PRISM framework indicates that patients’ characteristics such as gender, socioeconomic status (SES), language and culture should be considered, to maximise intervention effectiveness and reach important patient subgroups. Similarly, factors characterising providers such as organisational leaders and management, staff and culture can influence their ability to adopt and successfully practice an intervention.

#### Patient characteristics

BC patients are a highly heterogeneous group due to the diversity in the type and stage of the disease, as well as various patients’ characteristics. However, most of the decision-support interventions have been developed to reflect the specificity of therapy options (chemo, endocrine, surgery, reconstruction), compared to patients’ attributes (e.g. frailty, literacy), which can influence SDM and the use of tools [24, 34]. Only in a limited number of studies, patients’ needs related to age, SES and literacy in some cases, were considered early in the development and later in the delivery of interventions.

Age was an important factor determining active participation in the development of interventions, with one intervention specifically designed and targeted at older patients [20]. However, when comparison is made across age classes, studies found mixed results for active use. Older patients might seek active engagement but prefer verbal communication, and web-based tools were only slightly better used among younger patients [21, 34, 42]. Similarly, little attention was given to the socioeconomic background and literacy levels of patients, with merely one intervention addressing these aspects [29]. Studies found that these characteristics affect PtDA uptake in practice. Patients with lower SES preferred using shorter, illustrated paper-based interventions, such as picture option grids rather than option grids, and receiving the conversation aids from surgeons directly rather than ahead of their appointment [27]. Likewise, the literacy levels of patients were found to hinder the effective use of PtDAs in practice [41]. Literacy in many cases was tackled by developing content accessible to patients with reading skills at the 7^th^-8^th^ grade level [31, 37] or using readability guidelines [20].

Finally, all analysed studies in our sample reported women patients as their target population of the intervention. Although male patients can also suffer from BC, they were not observed in the available literature.

#### Provider characteristics

Organisations eager to adopt SDM interventions in standard care need to carefully consider the teams and professionals expected to use them daily. Though these factors can vary widely across organisations and healthcare cultures, we managed to identify two dominant themes regarding the organisational aspects of implementation: i) clinicians’ experience and attitude toward SDM and PtDAs and ii) teamwork [24, 33, 39]. Discrete strategies [16] can be used to boost the willingness of staff and clinicians to apply SDM interventions in practice.

Several studies stress that HCPs’ lack of motivation to deliver the tool [25], lack of endorsement and lack of competence at SDM and PtDA use might lead to inaccurate or limited use of such interventions [24, 34, 41, 48]. To tackle these issues, leadership buy-in is a good predictor of the sustained adoption of PtDAs. Identifying key clinicians (e.g. surgeons) and key personnel (e.g. informatics), who can familiarize others in the organisation and encourage the daily use of PtDAs, were important strategies for ensuring adoption [35, 42]. In Belkora et al. [38], a major success factor for implementation was the leadership’s willingness to subsidise staff participation in the programme by donating one day per week of each intern’s time. These leaders not only provide the necessary institutional support for adoption but contribute, in the long run, to the dissemination and thus the sustainability of SDM interventions [26, 27, 30].

To address professionals’ motivation, studies suggest fostering a shared vision and team commitment concerning the importance of collaborative deliberation with patients. Widespread adoption of such practices would guarantee consistency in the delivery and equitable services to all patients [24, 33]. Similarly, training was an important aspect to boost motivation which we discuss in the next section.

The last aspect concerns the role of multidisciplinary teams (MDT). Sometimes clinicians question the use of SDM, arguing that MDTs already guarantee that the best possible treatment is chosen. Yet, MDT’s treatment advice delivered in a clinical encounter seems to reinforce the uneven power balance between professionals and patients, becoming a barrier to SDM [24, 48]. On the contrary, there was value in using the PtDAs during MDT meetings or indicating the use of PtDA in the meeting report [19, 21]. These practices are prominent because other HCPs, besides clinicians facing patients, have an important role in contributing to SDM behaviour, and because they are easily transferable to other health settings. Currently, multidisciplinary teams are becoming the norm in BC settings. Thus, understanding their role both towards clinicians and patients can help design better strategies for SDM interventions’ uptake.

### Implementation and sustainability infrastructure

A carefully developed implementation plan is key to bridging the gap between theoretical research and medical practice. Implementation and sustainability infrastructure typically involves observing results and adjusting procedures accordingly, engaging designated teams, providing training and resources, developing protocols, and, more broadly, building a multilevel programme for long-term sustainability [10].

Among the first academic investigations on implementation, Silvia and colleagues [34, 35] emphasised infrastructural elements such as organisational flexibility (e.g. adaptable implementation procedures and methods of delivery), and staff well-informed about the intervention, to achieve a sustained adoption across clinical sites. Conversely, the lack of clinical and system support, scheduling problems and general scarcity of human and technical resources were deemed key infrastructural barriers.

In the selected studies, several infrastructural factors were associated with the effective implementation of decision-support tools. First, a crucial element were the *subjects* delivering the intervention. Thus, having designated teams [24], frontend staff such as senior residents in training [40], (specialised) nurses [42], coaches [33], and social workers or patient educators [34] proved valuable to support PtDA uptake. In Bruce et al. [42] web-based information was introduced over the phone by assigned nurses or breast centre navigators, either at the time of diagnosis or during the surgery clinic appointment. Similarly, in Belkora et al. [38], trained interns were engaged in calling patients to coach them on the use of PtDAs. In general, several studies underlined the critical role of cancer nurse specialists or decision coaches as an important link to bring cohesion within teams [33, 48], and for PtDA promotion to patients [24, 30].

Second, training and providing educational material were prevalent strategies highlighted in the studies. Training sessions on SDM rationale and use of PtDA concerned both patients and clinicians (e.g. oncologists, surgeons, radiologists), or other staff (e.g. nurses, coaches, senior residents). HCPs receiving training on tools were more likely to distribute them [41]. Besides the PtDA functioning, clinicians could receive more general instructions on SDM and communication skills, *inter alia* [27, 33, 40, 41, 47, 48]. For example, in the study of Savelberg et al. [24], an introductory meeting to explain the programme and a short video were designed to raise awareness regarding SDM processes and to provide the team with coaching, training, and on-the-job instructions. To stress the importance of training all team members and preparing them for new roles, Berger-Hoeger and colleagues [48] developed a training course for nurses and a workshop for clinicians.

Finally, the use of (conceptual) frameworks or standards was an effective strategy to foster SDM behaviour while sharing best practices. Van Veenendaal and colleagues [47] designed an implementation programme following a framework responding to barriers and facilitators at four implementation levels: innovation, users, organisational and socio-political context. Similarly, Bruce et al. [42] used the Replicating Effective Programs (REP) framework to develop the implementation strategy for delivering web-based information to patients before their surgical consultation. Overall, ten studies in the sample used or referred to a framework for drafting and supporting an implementation strategy (see Table 1).

Infrastructural barriers to implementation comprised the costs of developing and integrating SDM tools with electronic health registries and issues related to protecting and securing personal health information records [30]. Poor infrastructure at the clinical centres (e.g. lack of space, computers, printers, and internet connection) was another major obstacle [35], particularly for digital PtDAs [19]. Finally, extensive training does not guarantee the retention of necessary skilled staff for sustained PtDA use. Staff turnover or internal organisational changes might lead to a loss of expertise and consistency in applying a given intervention (idem).

### External environment

The need to adopt SDM interventions has been at the centre of policy developments. Various external pressures, including clinical guidelines or regulatory frameworks, can exert influence in diametrically opposite directions on the decision to adopt SDM interventions in clinical practice.

Increased interest from policy and regulatory actors can greatly assist SDM uptake. In the USA, the 2010 Affordable Care Act (ACA) contains provisions for using SDM in clinical practice to improve care outcomes [49]. To facilitate adoption, ACA issued guidelines for funding, developing, certifying, and implementing decision-support interventions in the US. Likewise, clinical guidelines can act as a key lever to foster the uptake of SDM and PtDAs. For instance, the European Society of Breast Cancer Specialists (EUSOMA) recommended that “each patient has to be fully informed about each step in the diagnostic and therapeutic pathway and must be given adequate time to consider the alternatives and make an informed decision” [48, 50]. To meet the EUSOMA requirements, the Dutch Breast Cancer Guidelines were built in the form of guidance-based clinical decision trees (CDT) to facilitate the evaluation of all possible treatment alternatives [51]. These authorities, together with patient associations and insurance companies, exert indirect pressure on health organisations to adopt decision-support interventions for quality certification [24].

Going beyond the hype around SDM approaches, a recent review of clinical practice guidelines found that they address SDM insufficiently and need improvement [52]. National or international recommendations can also backfire the implementation. National quality requirements, according to which cancer treatment should start within 5 weeks from diagnosis, were found to be too stringent for shared patient-clinician deliberation [24]. Other factors interfering with SDM behaviour were clinicians’ fear of legal consequences and compliance with guideline recommendations as a pre-requisite for centre re-certification [48].

Lastly, (non-profit) organisations, scientific associations, or member-based societies act as facilitators for the development and spread of SDM approaches. Notable examples are the Informed Medical Decisions Foundation (now Healthwise) which was among the first to issue PtDAs for different types of BC [38, 53], and the International Shared Decision-Making (ISDM) Society [54]. Recognised standards exist to guide the design, development, implementation and evaluation of PtDAs, like the International Patient Decision Aids Standards (IPDAS) and the Ottawa Decision Support Framework [55, 56]. In our pool of selected articles, more than 53% of the studies (corresponding to 9 interventions) explicitly used IPDAS recommendations.

## Discussion

The interest in embedding SDM in clinical encounters has pushed the academic and medical community to develop and pilot-test various decision-support interventions, including patient DAs. This review has investigated published empirical research on the adoption of SDM interventions in BC clinical practice. To date, much more research has evaluated the efficacy of SDM interventions on patient outcomes (knowledge, decision conflict, satisfaction) [31, 57–59] than evidence available to inform the uptake of these practices in routine care. In response to calls for research to follow a holistic approach to implementation [60], this analysis used the PRISM framework to identify factors at different levels and their interaction in the implementation process of SDM interventions.

Strategies supporting SDM behaviour can be complex interventions that affect the organisational, infrastructural, team and clinician-patient level components. It is encouraging that two-thirds of the 19 studies scrutinising these aspects were published since 2017, with more evidence available from Europe. Roughly half the selected studies used an implementation framework to guide the adoption of proposed interventions from a systemic perspective. However, the evidence from other papers did not necessarily reflect meticulously designed plans but rather the use of discrete strategies covering some PRISM domains. While the use of discrete strategies has the advantage of focusing on specific domains (clinical and administrative staff, patients) or processes (integration with EHR, workflow re-design), they can serve as ‘building blocks’ for formulating multilevel strategies [16]. Multilevel programmes such as the one developed by van Veenendal and colleagues [61, 62] can be seen as modular approaches that allow adaptation, customization and scalability to other care settings and contexts. More work and guidance are needed regarding which types of strategies are more likely to be effective in routine breats cancer settings.

The analysis revealed a growing demand from regulatory agencies and the professional/scientific community articulated through the provision of standards, checklists, frameworks, and guidelines. However, the degree of success of a strategy might vary depending on the context. Policies and accreditation criteria can affect both negatively and positively the decision of organisations to adopt practices supporting SDM behaviour [24, 48].Given the complexity of healthcare systems, more effort is necessary to move from regulations requiring patient-centred care to actively practicing it. Support from legislation can be supplemented with guidance about facilitating SDM in everyday care (e.g. NICE guidelines in the UK [6, 63]), or aligning rules and norms with the application of SDM [62]. Future studies could include system-level factors in their analysis, take a cross-country outlook, and focus on less researched contexts.

The review highlights the components of strategies at the organisational level. Specifically, having designated clinical staff, conducting training for all team members, and developing and distributing educational material are good predictors for uptake. However, all these studies lack a consensus on the role of (specialised) nurses and multidisciplinary teams in the actual practice of SDM interventions. On the latter, promising evidence shows that MDTs have an increasing role both towards single clinicians and by encouraging the use of PtDA during encounters; yet the evidence is still slender [19, 21, 61]. Related, the differences in healthcare systems limit our understanding of the role of (specialised) nurses or social workers. Further research would shed light on the feasibility of sessions with nurse-led and expert coaching, especially in contexts where these workers have a marginal role. In the future, combined strategies could be designed to better integrate different specialty clinicians in promoting deliberation (by revising their roles), using several incentive schemas, and providing practical training.

Co-creation with patients and health care providers (users) holds promise in accelerating the translation of SDM interventions into practice. As shown by some studies, co-developed interventions are more responsive to users’ needs and concerns and contribute to later acceptance and diffusion. Nevertheless, user engagement in the design, and later in implementation, is limited [61]. In the future, strategies should contemplate users, in particular patients, as part of both the development process and (more importantly), implementation activities [16]. Research is also needed to determine whether co-created practices are more likely to be adopted and sustained.

Given the increased importance of electronic systems and dissemination of digital PtDAs (37% of interventions), the topic of integration costs, interoperability, privacy and data management are still under-explored in the relevant literature. These aspects could prove highly relevant, especially in emerging contexts with fewer resources and less attention to such innovations. Further, focusing on infrastructural elements can prove useful for devising systems that identify and refer tools directly to patients, besides relying on clinicians.

While some of the findings discussed above find support in previous research [60], our analysis stresses the value of devising multifaceted strategies that cover both patients and providers, to favour a cultural shift towards SDM. In that sense, recent findings align with our focus on a whole-team approach [44], by engaging and (re)distributing tasks to (specialised) nurses and multidisciplinary teams [64], and offering interprofessional training [61, 62]. The review takes a step further to suggest that co-creating interventions with users is an opportunity to tackle distrust [57], offer better-fitting interventions and even supply evidence other than the traditional number of distributed tools. Compared to reviews covering decision aids generally (screening, tests, treatment) in various clinical areas [44, 60, 64], our contribution is specific to decision support after diagnosis in BC. We argue that the organisational and team implications are different in situations of treatment decision-making, requiring efforts both at the preparatory stage and during consultations, supported by specific staff training. Finally, we provide an integrated perspective on factors influencing the adoption and use of decision aids rather than focusing on barriers and facilitators found in prevailing research.

These findings are subject to several limitations. Although the use of PRISMA-ScR has enabled a structured and transparent review of the literature, the underlying data sources (peer-reviewed literature, official guidelines, regulatory documents, etc.) used for the selection of studies may be prone to bias. Further, only papers published in English were ultimately considered, possibly missing relevant studies in the field in other languages. Due to the heterogeneity of study designs, their quality and strength of evidence were not assessed, and no comparison of reported results was attempted. Related, our analysis did not consider the effect or success of implementation approaches used in the studies, mainly for two reasons. First, because information about reach was not consistently reported (e.g. how many patients use the tool adopted or how many hospitals/centres adopt a newly developed intervention outside the study context). Second, information was limited about the best strategy of implementation and how to maintain these interventions. Finally, although the interrater agreement for study selection was considerable, the second half of records was screened by only one reviewer.f Nevertheless, we are confident that our findings synthesised according to the PRISM framework can guide future considerations on successfully implementing decision-support interventions in BC care and highlight new avenues of inquiry in this field.

## Conclusions

Embedding SDM in BC care delivery is both appropriate and highly challenging. Hence, multilevel and multifaceted approaches are needed to consider the patient-provider dyad, teams, organisations and system-level factors. Future studies will have to distinguish between implementation strategies for the initial roll-out of interventions and those for their sustainable maintenance in the longer term. Finally, future research should go beyond the initial qualitative design to test implementation strategies through experimental or large-scale quantitative measures, enabling the transferability and scalability to other contexts and understanding of their relative efficacy.

### Supplementary Information

**Additional file 1.** Checklist of PRISMA-ScR guidance used for reporting results according to study design.

**Additional file 2.** Details of the search queries for PubMed, Web of Science and Scopus databases.

**Additional file 3.** Data extraction template for PRISM framework domains.

## Data Availability

All data generated or analysed during this study are included in this published article and its supplementary information files.

## Abbreviations

BC: Breast cancer
CPM: Contralateral prophylactic mastectomy
EHR: Electronic health record
HCP: Healthcare professional
IPDAS: International Patient Decision Aids Standards
MDT: Multidisciplinary teams
PRISM: Practical, Robust Implementation and Sustainability Model
PtDA: Patient Decision Aid
SDM: Shared decision-making
