# Supplementary material for: Implementing shared decision-making interventions in breast cancer clinical practice: a scoping review
Source: BMC Med Inform Decis Mak. 2023 Aug 23;23:164. doi: 10.1186/s12911-023-02263-8 (PMC10463920; doi:10.1186/s12911-023-02263-8)
Supplement: Supplementary file 2 — Additional file 2. Details of the search queries for PubMed, Web of Science and Scopus databases. [file 12911_2023_2263_MOESM2_ESM.docx]

*Table 2 Details of the search query in Pub Med, Web of Science and Scopus*

| **Pub Med** |
| --- |
| ((shared decision making[Title/Abstract]) OR (patient aid[Title/Abstract]) OR (patient decision tool[Title/Abstract]) OR (decision support[Title/Abstract])) AND (breast cancer[Title/Abstract]) |
| **Web of Science** |
| (TI=(decision aid OR shared decision making OR shared decision-making OR patient decision tool OR decision support) AND TI=(breast cancer)) OR (AB=(decision aid OR shared decision making OR shared decision-making OR patient decision tool OR decision support) AND AB=(breast cancer)) |
| **Scopus** |
| (TITLE-ABS ("shared decision making" OR "patient aid" OR "patient decision tool" OR "decision support") AND TITLE-ABS ("breast cancer") AND PUBYEAR > 2006 AND ( LIMIT-TO ( DOCTYPE,"ar" ) OR LIMIT-TO ( DOCTYPE,"cp" ) OR LIMIT-TO ( DOCTYPE,"re" ) OR LIMIT-TO ( DOCTYPE,"ch" ) OR LIMIT-TO ( DOCTYPE,"sh" ) ) ) |
