# Supplementary material for: Implementing shared decision-making interventions in breast cancer clinical practice: a scoping review
Source: BMC Med Inform Decis Mak. 2023 Aug 23;23:164. doi: 10.1186/s12911-023-02263-8 (PMC10463920; doi:10.1186/s12911-023-02263-8)
Supplement: Supplementary file 3 — Additional file 3. Data extraction template for PRISM framework domains. [file 12911_2023_2263_MOESM3_ESM.docx]

*Table 3 Data extraction template for PRISM domains*

| Reference (author, year | Intervention name | Development method | Development participants | | IPDAS (yes=1no=0) | Implementation strategy | Recipients | Challenges | | Infrastructure |
| --- | --- | --- | --- | --- | --- | --- | --- | --- | --- | --- |
|  |  |  | # | type |  |  |  | Patient | HCP |  |
